# Disseminated Cryptococcosis in a patient with idiopathic chylothorax and chylous ascites treated with a denver peritoneo-venous shunt: a case report and review of the literature

**DOI:** 10.22034/cmm.2025.345449.1689

**Published:** 2026-02-01

**Authors:** Kyotaro Kawase, Yoshitaka Wakabayashi, Shin Nakayama, Ai Yamamoto, Kiichi Watanabe, Masayoshi Yamamoto, Takatoshi Kitazawa

**Affiliations:** 1 Department of Internal Medicine, Teikyo University School of Medicine, Tokyo, Japan; 2 Department of Radiology, Teikyo University School of Medicine, Tokyo, Japan

**Keywords:** Denver peritoneo-venous shunt, Disseminated cryptococcosis, duodenal perforation, chylothorax, chylous ascites

## Abstract

**Background and Purpose:**

Disseminated cryptococcosis is an opportunistic infection in immunocompromised individuals. Chronic idiopathic chylothorax and chylous ascites can cause immunodeficiency via loss of immunoglobulin and lymphocytes in the late stage.

**Case presentation:**

This study aimed to present a case of disseminated cryptococcosis shortly after duodenal perforation in an immunocompetent patient with idiopathic chylothorax and chylous ascites that had been treated with a Denver peritoneo-venous shunt. Diagnosis of dissemination was confirmed by positive cryptococcal culture of blood, pleural effusion, ascites, and the removed shunt. The patient had low CD4+ lymphocytes and hypogammaglobulinemia at the onset of cryptococcosis.

**Conclusion:**

It should be noted that cryptococcosis can also occur in cases of idiopathic chylothorax and chylous ascites with low CD4+ or gamma globulin levels.

## Introduction

Disseminated cryptococcosis is an opportunistic infection that mainly occurs in immunocompromised hosts. In addition to Human immunodeficiency virus (HIV) infection, corticosteroid use, solid organ transplantation, and certain immunosuppressive agents are known risk factors for cryptococcosis [1]. Inhalation of *Cryptococcus* into the lungs is thought to cause hematogenous dissemination to the central nervous system [1]. In contrast, skin and soft tissue infections or visceral presentations, including gastrointestinal disease, are rare causes of disseminated cryptococcosis [2].

Chylothorax and chylous ascites are characterized by loss of chyle, which contains lymphocytes and fats [3, 4]. In the end stage of chylothorax and chylous ascites, lack of nutrition and a decrease in lymphocytes can lead to opportunistic infections, including cryptococcosis, although no large-scale studies have addressed the exact incidence. A small number of cases of idiopathic chylous ascites have also been treated with Denver shunts [5].

We encountered a case of disseminated cryptococcosis shortly after the treatment of idiopathic chylothorax and chylous ascites with a Denver shunt. To date, this is the first case report of cryptococcosis in a patient with chylothorax and chylous ascites treated with a peritoneo-venous shunt.

## Case presentation

A 57-year-old Japanese male with a 5-year history of idiopathic chylothorax and chylous ascites was admitted to our hospital for peritoneo-venous shunt placement. He had not responded to a fat-restricted diet therapy and had a medical history of lymphatic-venous anastomosis. It should be mentioned that he had no history of exposure to birds. On day 20 of hospitalization, pleural and ascites fluids were collected, and no growth was observed in aerobic and anaerobic cultures at our hospital. The peritoneo-venous shunt was implanted on day 22 of hospitalization.

However, 10 days after shunt implantation, the patient developed a fever of 38 °C, and *Klebsiella pneumoniae* was isolated exclusively from a right pleural fluid aspirate, for which cefotaxime administration was initiated. Blood, left pleural fluid, and ascitic fluid cultures were negative, and the serum IgG level was 780 mg/dL (normal range: 680-1620 mg/dL) at that point. The peritoneo-venous shunt was retained.

On day 55, the patient complained of a sudden onset of upper abdominal pain with signs of peritoneal irritation. Contrast-enhanced computed tomography showed wall discontinuity in the duodenal bulb and free air over the gallbladder bed, suggesting duodenal bulb perforation (Figure 1A, B). Surgery was not performed due to the low serum albumin level of 1.5 mg/dL (normal range: 4.1-5.1 mg/dL); therefore, the patient received conservative treatment. Cefotaxime was changed to piperacillin/tazobactam and micafungin.

**Figure 1:**
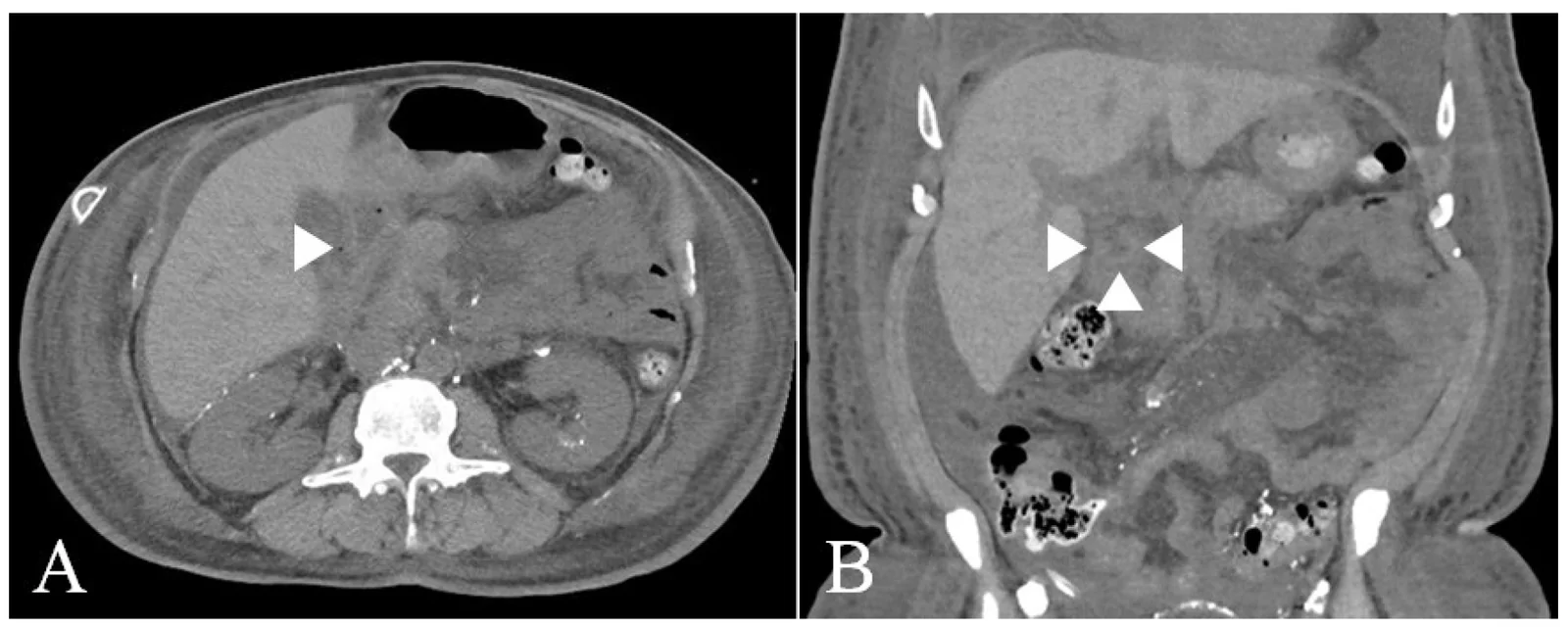
Computed tomography scan of the abdomen on day X. **A.** Horizontal section shows free air at the side of the duodenal bulb. **B.** Coronal section shows irregularity of the duodenal wall.

The next day, he developed a fever, and a blood culture was performed, which became positive for yeast-like fungi at 69 h-a relatively prolonged time for *Candida* species (Figure 2). Although *Candida* species was initially considered to have originated from intestinal perforation, a multiplex polymerase chain reaction test (FilmArray^®^, bioMérieux, Tokyo) detected the *Cryptococcus neoformans* gene. Moreover, internal transcribed spacer region sequencing of the blood culture identified *Cryptococcus neoformans* variant *grubii* (GenBank accession number: NR_130682.1). The minimal inhibitory concentrations of the strain were 0.5, 2, and 2 µg/mL for amphotericin-B, flucytosine, and fluconazole, respectively, based on Clinical and Laboratory Standards Institute (CLSI) M27-Ed4, 2017. The CD4+ lymphocyte count was 36/µL, the serum IgG level was 465 mg/dL, and anti-HIV antibodies were negative on day 59.

**Figure 2:**
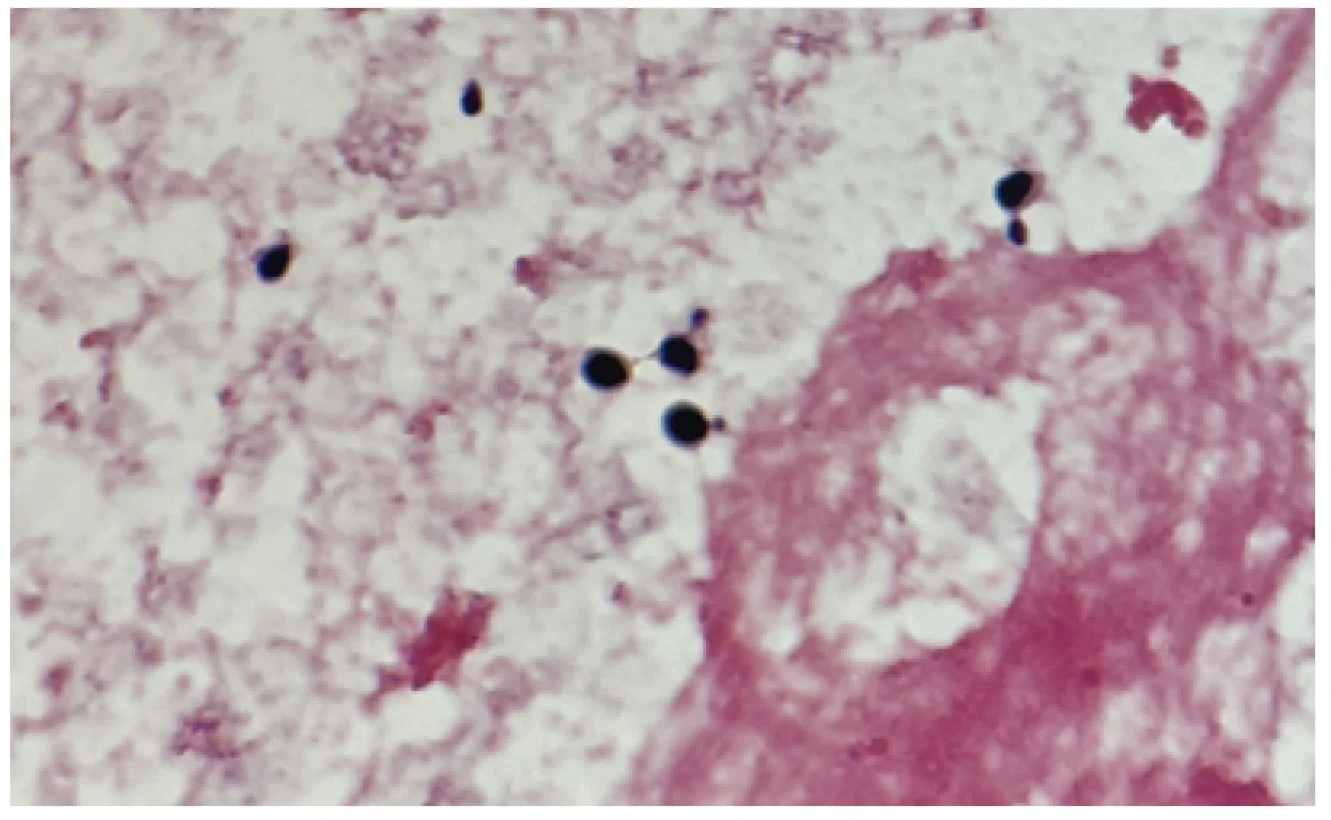
Gram-staining of the shunt tip indicates the presence of yeast-like fungi.

Hence, the peritoneo-venous shunt was removed, and combination antifungal therapy was started with liposomal amphotericin B (200 mg q24h, 3 mg/kg ideal body weight/dose) and fluconazole (500 mg q24h, 12 mg/kg ideal body weight/dose adjusted for renal function) to treat the disseminated cryptococcosis on day 59. The patient also had low blood pressure, and meropenem and teicoplanin were initiated, considering the possibility of bacterial sepsis. Thereafter, *Cryptococcus neoformans* was detected in cultures of the removed shunt, bilateral pleural and ascitic effusions, and sputum. Follow-up blood cultures remained positive on days 62 and 64; however, the culture obtained on day 69 was negative. Although the patient was successfully weaned off the catecholamines, type 2 respiratory failure gradually became more pronounced, and he died on day 83 of hospitalization.

## Discussion

Disseminated cryptococcal disease is most commonly associated with HIV infection. However, it has also been found in other types of immunocompromised HIV negative patients, such as those with solid organ transplantation, liver cirrhosis, and long-term use of immunosuppressive agents [6, 7]. As is known, cellular immunity, especially CD4+ lymphocytes and macrophages, plays an important role in host immunization against cryptococcal infection [1]. Humoral immunity also seems to be a key component. Upon binding with immunoglobulin G-based immune complexes comprised of antiglucuronoxylomannan antibody-opsonized *C. neoformans*, the Fcγ receptor III, along with raft-forming cholesterol, initiates the signaling cascade associated with phagocytosis [8]. There have been several case reports of disseminated cryptococcosis in patients with low immunoglobulin G levels [9, 10]. Lower-than-expected levels of antibodies in patients with disseminated fungal infection are often the result of the presence of underlying diseases or the use of immunosuppressive drugs in patients. Disseminated cryptococcosis should also be considered in the differential diagnosis in immunocompromised cases of unspecified multifocal infection with low immunoglobulin G levels.

A previous study analyzing the risk factors for disseminated cryptococcosis did not include chylothorax and chylous ascites [11], although both conditions are believed to be associated with immunodeficiency [5, 12]. Previous reports have described T-cell or immunoglobulin depression in postoperative chylothorax patients and children with chylothorax [13-15].

A case of disseminated cryptococcosis with a background of chronic chylothorax was also reported by Mundo et al. [16]. Both their case and the present one appear to have developed disseminated cryptococcosis against a background of humoral and cellular immunodeficiency, although disseminated cryptococcosis can occur in immunocompetent individuals [17]. The patient in the present study had CD4+ lymphocyte depression and hypogammaglobulinemia, although the measurements were made after the onset of cryptococcosis. This suggests that measurement of immune status might help to estimate the risk of opportunistic infections in patients with chylothorax and chylous ascites.

Shunt infection is known as a potentially critical complication of Denver shunts. In a study of patients with refractory ascites undergoing Denver shunt implantation, 4.8% of them developed infection as a postoperative complication [13]. Treatment of shunt infection requires shunt removal for source control, although it is sometimes recurrent and refractory [13]. In the case reported by Mundo et al., a pleuro-venous shunt was transplanted 3 months before the development of disseminated cryptococcosis. In that case, cryptococci were detected at the tip of the shunt when the shunt was removed [16]. In the present case, early removal of the shunt and initiation of antifungal therapy for disseminated cryptococcosis likely resulted in resolution of the fungemia.

Cryptococcal peritonitis is a rare entity and is known to present as spontaneous peritonitis in cirrhotic patients or peritoneal dialysis catheter-related infection [18]. In the present case, cryptococcemia was detected shortly after duodenal perforation. In fact, ascitic culture and blood culture were negative one month before perforation, and bilateral pleural culture was negative 10 days before perforation. It is possible that duodenal cryptococcal infection in this case resulted in secondary peritonitis, which led to fungemia and empyema via the peritoneo-venous shunt. Although primary gastrointestinal cryptococcosis is rare, there are some case reports of gastrointestinal cryptococcosis complicated with intestinal perforation [19-21]. In the present case, since endoscopic and pathological examination of the duodenum was not performed due to the risk of bleeding or worsening perforation, it was not possible to determine whether duodenal lesions caused by cryptococci were present or whether the infection was the cause of the perforation. Latent primary pulmonary cryptococcosis might also have evolved into dissemination, although this was less likely due to the lack of pulmonary symptoms or specific findings prior to perforation.

The patient in the present study had a complicated gastrointestinal perforation at the time of diagnosis of cryptococcosis, and since oral administration of flucytosine was not possible, he was treated with a combination of liposomal amphotericin B and intravenous fluconazole. Although we referred to the guidelines for HIV-infected patients in the treatment of disseminated cryptococcosis [22, 23], we believed that the choice was reasonable even in a non-HIV-infected immunocompromised patient.

## Conclusion

This study presented a case of cryptococcal fungemia, peritonitis, and empyema in a patient treated for idiopathic chylothorax and chylous ascites with a Denver peritoneo-venous shunt. Eventually, *Cryptococccus* was identified, antifungal medications were administered, and the shunt was removed, but without improvement. Although cases of disseminated cryptococcosis in non-HIV-infected patients are rare, patients with low CD4+ lymphocytes and hypogammaglobulinemia are at risk for developing the disease. Hence, caution is advised in cases of chylothorax and chylous ascites, since they are also at risk of developing cryptococcosis.
